# Neuroprotective Strategies for Stroke by Natural Products: Advances and Perspectives

**DOI:** 10.2174/1570159X21666230717144752

**Published:** 2023-09-01

**Authors:** Aifen Liu, Jingyan Hu, Tzu-Shao Yeh, Chengniu Wang, Jilong Tang, Xiaohong Huang, Bin Chen, Liexiang Huangfu, Weili Yu, Lei Zhang

**Affiliations:** 1 Institute of Interdisciplinary Integrative Medicine Research, School of Medicine, Nantong University, Nantong 226001, China;; 2 Department of Nutrition and Food Hygiene, School of Public Health, Nantong University, Nantong 226019, China;; 3 Department of Pharmaceutical Botany, School of Pharmacy, Naval Medical University, Shanghai 200433, China

**Keywords:** Ischemic stroke, natural products, neuroprotection, antioxidation, mitochondrial dysfunction, ER stress, apoptosis, inflammation

## Abstract

Cerebral ischemic stroke is a disease with high prevalence and incidence. Its management focuses on rapid reperfusion with intravenous thrombolysis and endovascular thrombectomy. Both therapeutic strategies reduce disability, but the therapy time window is short, and the risk of bleeding is high. Natural products (NPs) have played a key role in drug discovery, especially for cancer and infectious diseases. However, they have made little progress in clinical translation and pose challenges to the treatment of stroke. Recently, with the investigation of precise mechanisms in cerebral ischemic stroke and the technological development of NP-based drug discovery, NPs are addressing these challenges and opening up new opportunities in cerebral stroke. Thus, in this review, we first summarize the structure and function of diverse NPs, including flavonoids, phenols, terpenes, lactones, quinones, alkaloids, and glycosides. Then we propose the comprehensive neuroprotective mechanism of NPs in cerebral ischemic stroke, which involves complex cascade processes of oxidative stress, mitochondrial damage, apoptosis or ferroptosis-related cell death, inflammatory response, and disruption of the blood-brain barrier (BBB). Overall, we stress the neuroprotective effect of NPs and their mechanism on cerebral ischemic stroke for a better understanding of the advances and perspective in NPs application that may provide a rationale for the development of innovative therapeutic regimens in ischemic stroke.

## 1 INTRODUCTION

Stroke is the second largest cause of death and a leading cause of disability globally [1]. Stroke can be classified into acute ischemic stroke (AIS) and intercellular hemorrhagic (ICH), and more than 80% are ischemic stroke that principally results from the occlusion of a cerebral artery and insufficient blood supply and is defined as infarction of the brain [2, 3], whose severity is related to the time of cerebral ischemia and the depth of the ischemic site. Currently, the best-evident treatment guidelines include intravenous thrombolysis and endovascular thrombectomy [4]. Intravenous tissue plasminogen activator (tPA) has become the only medication approved by FDA for AIS and can salvage dying cells from the ischemic penumbra, but it should be administered within 4.5 h of stroke onset, and the delayed tPA infusion would increase the hemorrhagic transformation [4, 5]. Endovascular thrombectomy can diminish disability in a wide group of patients with large vessel occlusion but should be performed within 6 h of stroke onset [4, 6]. With rapid reperfusion, the penumbral brain can be salvaged and resume normal function. However, the treatment of ischemic stroke is time-critical and remains one of the key challenges to maximizing the benefits of these therapies, so it is necessary to explore their full therapeutic potential.

The ischemic brain is grouped into the irreversibly damaged infarct core and the surrounding ischemic penumbra. The infarct core lies in the central zone of the infarction area and consists of dead or dying tissue, whereas the ischemic penumbra is located around the infarct core and can be salvaged through early reperfusion [4, 7]. Shortly after the injury, neurons in the penumbra activate survival signaling pathways, which remain active for hours to days [8]. If the ischemia persists in cerebral tissues for a prolonged period of time, the infarct core will probably extend to the penumbra [8]. Subsequently, neurons in other areas of the brain will also die because of the loss of contact with ischemic neurons (called secondary neuronal injury). Therefore, it is essential for neuroprotective therapy to salvage penumbral neurons [7].

Neuroprotection was gradually studied from the 1970s to 1990s, developed during the 2000s, and has received significant attention over the past 40 years [9]. For acute ischemic stroke, neuroprotection can be characterized as approaches, applied alone or in combination, that directly or indirectly target the brain parenchyma with the aim of antagonizing the harmful molecular and cellular events caused by ischemia, allowing brain cells to survive and the penumbra to be spared. As the ischemic cascade of stroke is a multifaceted and complex pathophysiological process, the multi-targeted neuroprotectants (cocktail therapy) may be promising to salvage the brain tissue of patients after successful revascularization in ischemic stroke [10].

Natural products (NPs), defined as small molecule compounds derived from natural sources, such as plants, animals, and microorganisms, have been used to treat human diseases for thousands of years. They are proven to be a valuable source of new drugs [11]. NPs are characterized by enormous scaffold diversity and structural complexity. Compared with synthetic compound libraries, NPs generally have a higher molecular weight, including a more important number of sp3 carbon and oxygen atoms, but fewer nitrogen and halogen atoms, more H-bond acceptors and donors, lower calculated octanol-water partition coefficients (clog values, indicating higher hydrophilicity), and greater molecular rigidity [12-14]. These differences may be beneficial.

First of all, the higher rigidity of NPs can be valuable for tackling protein-protein interactions in drug discovery [15]. Secondly, NPs are structurally modified to serve particular biological functions, such as the regulation of endogenous defense mechanisms and competitive activity with other organisms [16]. Thirdly, the NP pool is enriched with ‘bioactive’ compounds, which cover a wider chemical space than standard synthetic small-molecule libraries [17]. Therefore, the rich structural diversity and complexity have prompted the production of NPs and derivatives with therapeutic applications [14]. So far, NPs have become valuable tools for developing front-line drugs, especially for cancer and infectious diseases [18, 19], as well as in other therapeutic areas, including cardiovascular diseases and multiple sclerosis [16, 20, 21]. Moreover, NPs could also improve microcirculation in the brain, protect from ischemic reperfusion (IR) injury by reducing oxidative stress and neuroinflammation, and modulate microglia polarization [10, 22, 23]. These reports support the role of the neuroprotection of NPs in ischemic stroke.

Here, we take into account the pathophysiological mechanism of stroke in detail and propose the prospect of NPs application in stroke management. This review summarizes the structure and function of diverse NPs, including flavonoids, phenols, terpenes, lactones, quinones, alkaloids, and glycosides in cerebral ischemic stroke. The involved pathophysiology mechanism of NPs in cerebral ischemia is also reviewed, which has neuroprotective effects against oxidative damage after stroke by targeting pathways upstream from the production of ROS, thereby diminishing their downstream effects on macromolecules [23, 24]. Acute stroke is followed by secondary neuroinflammation, which leads to cell death. The proinflammatory signals secreted by immune cells activate resident cells and attract peripheral inflammatory cells, which infiltrate the lesion area [25, 26]. Some natural compounds extracted from natural medicinal formulations reduce neuroinflammation during the acute stage of stroke [22]. Furthermore, immediately after ischemic stroke, the activation of microglia polarizes into either the classic (M1) or the alternative (M2) activation phenotype [27, 28], and some NPs regulate microglia polarization and play neuroprotective roles in stroke [29]. Finally, disruption of the blood-brain barrier (BBB) plays a crucial role in developing neurological dysfunction after cerebral ischemia. NPs protected by BBB appear to be a promising strategy for treating ischemic stroke [23]. Among them, the most important aspect is that ischemic stroke produces excessive reactive oxygen species (ROS) that lead to oxidative stress, then induces widespread damage by oxidizing lipid acids, proteins, and DNA, and ultimately leads to intrinsic and extrinsic apoptosis [30], necroptosis, autophagy, ferroptosis, parthenotes, phagocytosis, and pyroptosis. Therefore, this critical review summarizes the latest findings in pharmacological research on neuroprotective targeted therapy and the emerging drug targets of NPs in ischemic stroke.

## 2 PHARMACOLOGICAL IMPORTANCE OF NPS IN CEREBRAL ISCHEMIC STROKE

Currently, various NPs have been utilized due to their unique advantages in the pathophysiological mechanism of ischemic stroke. In this article, existing evidence indicating the neuroprotective effect of these NPs in cerebral ischemic stroke models is described (Table **1**).

### 2.1 Flavonoids

Flavonoids are plant-derived natural products that are beneficial to human health [31]. This class of molecular compounds can undergo modifications in aromatic cycles, including hydroxylation, methylation, glycosylation, acylation, or prenylation. Many of the biological effects of flavonoids are attributed to their potential cytotoxicity and antioxidant abilities. The oxidation of flavonoids in planta is mainly catalyzed by polyphenol oxidases (catechol oxidases and laccases) and peroxidases. Flavonoids act as scavengers of free radicals, such as reactive oxygen species (ROS), and prevent their formation by chelating metals [32]. These activities are induced by environmental stresses like pathogen attacks. Their complex mechanism of action is regulated at several levels, involving transcriptional to post-translational mechanisms and the differential subcellular compartmentalization of enzymes and substrates [32]. Extensive studies have provided information on various activities of flavonoids, including antimicrobial, antioxidant, and anti-inflammatory activities [33]. Neuroprotection of flavonoids in cerebral stroke is garnering attention worldwide. Flavonoids, such as baicalin [34-42], puerarin [43-45], icariin [44], breviscapine [46, 47], quercetin [48, 49], scutellarin, scutellarein [50-52], apigenin [53, 54], and vitexin [55, 56], have been extensively studied for their multi-functions (Table **1**). Some NPs have shown antioxidant, anti-inflammatory, and anti-apoptotic properties in *in vivo* and *in vitro* studies on the prevention and treatment of stroke. The signaling pathways involved are PI3K/AKT/mTOR and NF-κB pathways [33].

### 2.2 Polyphenols

Polyphenols are common nutrients derived from fruits, vegetables, tea, coffee, cocoa, mushrooms, beverages, and traditional medicinal herbs. They are potential substances against oxidative-related diseases, and the strong radical-scavenging properties of polyphenols can exhibit antioxidative and anti-inflammation effects. Polyphenols reduce ROS production by inhibiting oxidases, superoxide production, and platelet aggregation and improving mitochondrial oxidative stress [57]. Polyphenols also weaken the glutamate-induced activation of calpains, normalize the level of cytosolic Bax and inhibit the release of AIF from mitochondria [58], MMP-9 downregulation [59], as well as mitochondrial dysfunction [60]. Polyphenols, such as calycosin [56, 61], resveratrol [62-65], curcumin [42, 66-69], salvianolic acid B [70-72], tanshinone IIA (TSA) [73-75], salvianolic acid A (SAA) [76-79], geraniin [80], and ellagic acid [81], play neuroprotective roles in ischemic stroke.

### 2.3 Terpenoids

Terpenoids are the largest class of NPs, most of which are derived from plants. These terpenoids are composed of five carbon isoprene units. They are grouped into three categories: sesquiterpenoids (artemisinin, parthenolide), diterpenoids (oridonin, triptolide), and triterpenoids (alisol, betulinic acid, oleanolic acid, platycodin D, and ursolic acid). Many terpenoids affect epigenetic and cellular death mechanisms by targeting NF-kB, Janus kinases-signal transducer and activated transcription proteins (JAK-STAT), activator protein-1 (AP-1), metalloproteinases (MMPs), DNA topoisomerase I and II, blocking the endoplasmic reticulum (ER) calcium ATPase pump, inhibiting proteasome, activating p53, and modulating DNA minor grooves [82, 83]. Furthermore, they have numerous biological properties, including anti-proliferative, apoptotic, anti-angiogenic, and anti-metastatic activities. They are also involved in the induction of autophagy *via* complex signaling pathways, such as MAPK/ERK/JNK, PI3K/AKT/mTOR, AMPK, NF-kB, and reactive oxygen species [83]. The primary mechanism of cell death induced by terpenoids is apoptosis [84]. Terpenoids, such as ginkgolide B [85-89], catalpol [90-93], celastrol [94-97], totarol [98], 2,3,5,4′-tetrahydroxystilbene-2-O-β-D-glucoside (TSG) [99-102], and phycocyanobilin (PCB), play neuroprotective roles in ischemic stroke.

### 2.4 Lactones

Lactones are cyclic esters of hydroxy acids. Hydroxy acids or their activated forms are typically the natural precursors in the biosynthesis of lactones. Cyclization makes it easier for drugs to pass through lipophilic membranes without transporters. Furthermore, the hydroxy acid is deprotonated mainly at the physiological pH level, further increasing the hydrophilicity and decreasing the vapor pressure [103]. Britanin ameliorates cerebral ischemia-reperfusion injury by inducing the Nrf2 protective pathway [104]. Andrographolide (ANDRO), a bitter diterpene lactone found in Andrographis paniculata (Burm.f.) Nees, possesses several biological effects, such as antioxidant, anti-inflammatory, and organo-protective effects. Some studies have shown that ANDRO (0.1 and 1 mg/kg i.p) decreased the infarct volume and neurological deficits and drastically lowered microglia cells and inflammatory response in permanent middle cerebral artery occlusion- (pMCAO-) or MCAO-induced rat model [105]. Similarly, targets and involved signal pathways in lactones, such as taraxasterol [106], britanin [104], bilobalide [107-109], and ligustilide [110-115], are shown in Table **1**.

### 2.5 Quinones

Quinones are privileged chemical structures that play crucial roles as redox and alkylating agents in various cell processes [116]. Quinone and naphthoquinone exert cytotoxicity *via* ROS or protein alkylation-induced damage. Paradoxically, some prenylated quinones possess antioxidant properties by scavenging DPPH and/or hydroxyl radicals [117]. Emodin (1,3,8-trihydroxy-6-methylanthraquinone) is a naturally occurring anthraquinone derivative found in the roots and barks of some plants, particularly in all kinds of Chinese herbs, *e.g*., Rheum palmatum (rhubarb), Polygonum cuspidatum (Japanese knotweed), Polygonum multiflorum [4] and several species of fungi (*e.g*., Pyrenochaeta terrestris and Aspergillus aculeatus). Furthermore, emodin exhibits multiple biological activities, such as antibacterial, anti-inflammatory, anti-metastatic, and immunosuppressive effects [118-121].

### 2.6 Alkaloids

Alkaloids are an important class of small NPs. Plants are estimated to produce 12,000 different alkaloids as secondary metabolites found in about 20% of plant species [122]. Compounds that contain heterocyclic moieties often show improved solubility and can facilitate the salt formation, which is important for oral absorption and bioavailability. Due to the characteristics of polymorphism, structural complexity, availability from natural sources, and generally low toxicity in normal cells, heterocyclic alkaloids and their derivatives have great potential to serve as G-quadruplex ligands through high throughput screening and other specific methods [123]. Several classes of isoquinoline alkaloids have been reported in this genus, including aporphine, protopine, protoberberine, tetrahydroprotoberberine, benzo[c] phenanthridine, phthalideisoquinoline, benzylisoquinoline, morphinan, and spirobenzylisoquinoline. These alkaloids exhibit various biological properties, such as analgesic, anti-inflammatory, anti-cancer, acetylcholinesterase, and butyrylcholinesterase inhibitory activities [124]. Eight new alkaloids, and three new naturally occurring alkaloids, together with three already known ones, were isolated from the bulbs of *C. decumbens*, which is a commonly used traditional Chinese medicine for treating post-stroke hemiplegia [124]. It has been observed that alkaloids, such as tethamethylpyrazine [125-128], leonurine [129-131], and berberine [132-135], play neuroprotective roles in ischemic stroke (Table **1**).

### 2.7 Glycosides

Glycosylation of proteins and lipids is critical to many life processes. NPs (secondary metabolites), such as flavonoids, steroids, triterpenes, and antibiotics, are modified with saccharides catalyzed by glycosyltransferases. Further modifications to the glycosides, such as acylation, oxidation, and degradation, also occur frequently. The resulting glycosides, including glycolipids, phenolic glycosides, steroid glycosides, and triterpene glycosides, have diverse structures and functions, some of which have pharmacological significance [136]. Astragaloside IV (AIV) is a natural triterpenoid saponin in the root of *Astragalus membranaceu.* It exhibits a wide variety of pharmacological effects, such as anti-oxidation stress, anti-inflammation, and anti-apoptosis, through multiple signals in cerebral ischemic stroke. This shows that astragaloside IV can reduce neuronal apoptosis and parthanatos in ischemic injury by preserving mitochondrial hexokinase-II [137]. Glycosides, such as astragaloside IV [70, 138-141], gastrodin [142], ginseng Rg1 [143], and salidroside [144], also play neuroprotective roles in ischemic stroke.

### 2.8 Phenylpropanoids

Phenylpropanoids and their derivatives are plant secondary metabolites widely present in fruits, vegetables, cereal grains, beverages, spices, and herbs [145]. They exert multifaceted effects on antimicrobial, antioxidant, anti-inflammatory, antidiabetic, and anticancer activities and play reno-protective, neuroprotective, cardioprotective, and hepatoprotective roles. They have low toxicity and a wide array of bioactive properties and are usually applied to human disease management [145]. In the embolic cerebral ischemic rat, p-CoA elevated NRF-1 levels have been reported to improve mitochondrial function. Phenylpropanoids, such as ginsenoside Rb1[146], ginsenoside Rc [147], ginsenoside F1 [148], α-Asarone [149, 150], β-asarone [151-153], ferulic acid [154], and caffeic acid [155], also play neuroprotective roles in ischemic stroke.

## 3 NEUROPROTECTION TARGETING TO OXIDATIVE STRESS IN ISCHEMIC STROKE

### 3.1 Inhibiting Excitotoxicity and Calcium Overload in Ischemic Stroke

Glutamate is a principal excitatory neurotransmitter in the central nervous system. During cerebral ischemia, injured neurons and activated astrocytes release glutamate through Ca^2+^- dependent exocytosis.

**Fig. (1):**
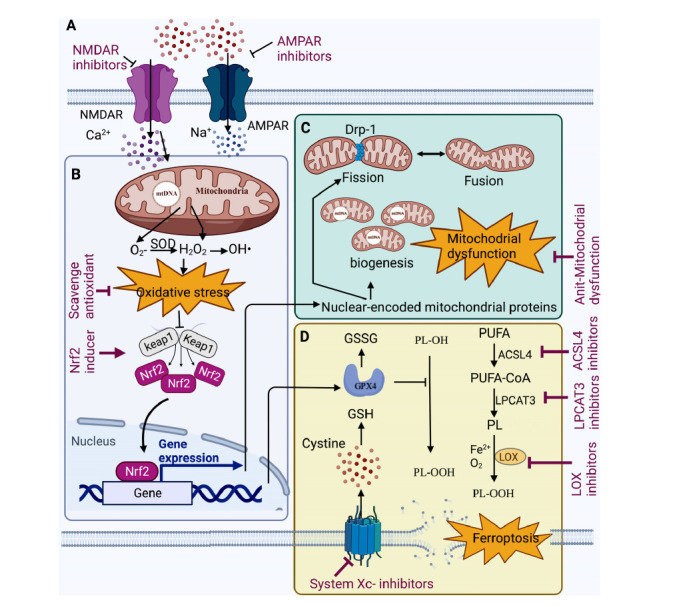
Schematic illustration of neuroprotection targeted to oxidative stress and induced mitochondrial dysfunction and ferroptosis in ischemic stroke. (**A**) NMDAR and AMPAR inhibitors targeted to reduce calcium overload. (**B**) ROS scavenger antioxidant and Nrf2 inducer in regulating the Nrf2 pathway under oxidative stress conditions. Disruption of the Nrf2-Keap1 association is mediated by free radicals, or inducers of Nrf2, and leads to a diminished rate of proteolysis, thereby enhancing the nuclear accumulation of Nrf2 in the nucleus. Nrf2 binds with AREs in the promoter region of its target genes and induces nuclear-encoded mitochondrial proteins and GPX4, resulting in an adaptive response. (**C**) Mitochondrial homeostasis is maintained through balanced biogenesis of new mitochondria, mitochondrial fission, and fusion. The mitochondrial shape is fundamental to mitochondrial (metabolic) activity. The efficiency of ATP production is increased, and the exchange of matrix content is favored in fused organelles. In contrast, fragmented organelles produce more reactive oxygen species (ROS) and are efficiently cleared by mitophagy. Mitochondrial fission is a hallmark of the early steps of apoptosis. **D.** The indicated pathways regulating ferroptosis sensitivity.

In addition, astrocytes also release glutamate through the glutamate-releasing SWELL1 Channel [156]. Then, postsynaptic glutamate receptors (excitotoxicity), including NMDAR and AMPAR, are overactivated and lead to intracellular Ca^2+^ overload and delayed neuronal cell death, which can be reversed by NMDAR or AMPAR antagonist (Fig. **1A**) [157, 158].

Intracellular Ca^2+^ buffering and storage is a neuronal feature that maintains a low intracellular calcium concentration ([Ca^2+^] i) compared to the extracellular space. Following ischemia, the [Ca^2+^] i can reach mM level because of the breakdown of these mechanisms, and the intracellular calcium overload is linked with the overactivation of enzymes, such as proteases, phospholipases, and endonucleases, which leads to the breakdown of proteins, lipids, and nucleic acids, and the final death of neurons [159]. Calpains are a group of Ca^2+^- dependent proteases contributing to the cleavage of plasma membrane proteins, synaptic vesicle protein transporters, mitochondrial proteins, and many other substrates [157]. Lipases and nitric oxide synthase (NOS) are also activated by calcium and then increase the production of free radical species [160]. Moreover, in addition to anoxic and ischemic cell death, the activation of transient receptor potential melastatin (TRPM) channels, such as TRPM7 and TRPM2, has been associated with oxidative stress [161-163]. Gualou Guizhi decoction (GLGZD), a classical Chinese medicine compound prescription, reverses brain damage with cerebral ischemic stroke, multi-component directed multi-target to screen calcium-overload inhibitors using the combination of molecular docking and protein-protein docking [164].

### 3.2 Scavenging Oxygen-Free Radicals and Antioxidation in Stroke

During cerebral IR, acute oxidative stress is induced and triggers severe tissue damage. Oxidative stress arises from the strong cellular oxidation potential of excess reactive oxygen species (ROS). The term ROS contains oxygen free radicals, such as superoxide anion radical (O_2_·), hydroxyl radical (·OH), nonradical oxidants, hydrogen peroxide (H_2_O_2_), and singlet oxygen (^1^O_2_). Most of the O_2_· is produced in mitochondria through the electron transport chain and electron leakage of the Krebs cycle; O_2_· is also produced by metabolic oxidases, including NADPH oxidase and xanthine oxidase. Excess O_2_· reduces transition metal ions, such as Fe^3+^ and Cu^2+^, reacting with H_2_O_2_ to produce ·OH through the Fenton reaction. ·OH is the strongest of the oxidant species and reacts indiscriminately with nucleic acids, lipids, and proteins [165]. Nitric oxide (NO), another ROS, functions as a neurotransmitter and is essential for dilating blood vessels [166].

In the event of cerebral ischemia, the associated brain damage is caused by excessive ROS *via* the following processes [23, 167]: (1) inhibition of protein synthesis, along with the damage of the DNA structure; (2) damage of the mitochondrial structure, which reduces the energy production; (3) lipid peroxidation (LPO) of unsaturated fatty acids in cell membranes and phosphoric acid degradation, further getting involved in the ferroptosis; (4) damage of endothelial cells, causing microcirculation disorder of the brain and increasing blood-brain barrier (BBB) permeability. Thus, it can be seen that stroke-induced oxidative stress is mainly associated with excessive ROS production, and the primary approach for antioxidant therapy for stroke is to reduce ROS production, scavenge existing ROS, and promote antioxidant defense. For example, Momordica charantia polysaccharide (MCP), an important bioactive compound of Momordica charantia, has neuroprotective effects on cerebral I/R injury by scavenging superoxide (O_2_·), nitric oxide (NO), and peroxynitrite (ONOO^−^), and inhibiting the JNK_3_ signaling pathway in the ischemic brain [168].

The nuclear erythroid 2-related factor 2/antioxidant response element (Nrf2/ARE) pathway is vital to regulate cytoprotective genes and enzymes in response to oxidative stress and treatment with NPs. Nrf2 is released from keap1 and translocated into the nucleus to express phase II cytoprotective genes and enzymes. Phosphorylation of Nrf2 also plays a critical role in the transactivation of antioxidant enzymes. NPs-derived inhibition of the Nrf2/ARE pathway has emerged as a promising strategy against multi-drug resistance, which can improve the therapeutic effect [169, 170].

Several Nrf2 inducers from NPs are effective in both *in vivo* and *in vitro* models of neurological disorders. Most of the NP-derived Nrf2 modulators are Michael acceptors, oxidizable phenols and quinones, isothiocyanates, dithiole, thiones, polyenes, or vicinal mercaptans. The only common feature of these compounds is the reaction with sulfhydryl groups by alkylation or oxidation, while the common property of most known ARE inducers is electrophilic quinones upon auto-oxidation. Diphenol is oxidized to its quinone derivative and then reacts with Keap1 in a Michael addition reaction with the corresponding orthoquinone (or paraquinone) form [170]. Totarol, isolated from the sap of Podocarpus totara, up-regulates the protein levels Akt, Nrf2, and heme oxygenase-1 (HO-1), increases the activity of GSH and SOD, suppresses oxidative stress, and produces a neuroprotective effect on stroke [98].

Taraxasterol and Z-ligustilide also significantly regulate the Nrf2 signaling pathway and reduce ROS production [106]. A new Nrf2 activator, britanin, plays the neuronal protective role by exerting its antioxidant activity by selectively binding to the conserved cysteine residue 151 of Keap1, which inhibits the ubiquitylation of Nrf2 [171]. To date, various NPs reportedly provide neuroprotection against oxidative damage after stroke. Most are natural antioxidants that regulate oxidative stress-related signaling pathways (Fig. **1B**) [23].

### 3.3 Mitochondrial Dysfunction-Targeting Therapies of NPs in Stroke

Mitochondria can efficiently generate ATP and are considered the ‘powerhouses of the cell’ [172]. Mitochondrial damage is a hallmark of ischemic stroke [4, 173-175], which is involved in several complex cellular processes beyond cell death, ranging from autophagy to stem cell differentiation and regulation of immune response [172]. NPs act on mitochondria in terms of modulation of biogenesis, dynamics, bioenergetics, calcium homeostasis, membrane potential, and inhibition of the oxytosis/ferroptosis pathway [11].

Mitochondrial biogenesis is impaired with aging and stroke [176]. One widely studied neuroprotective compound through modulation of mitochondrial biogenesis signaling is the polyphenol resveratrol from the berries of Vaccinium species and other plants. Mitochondrial dynamics are crucial to regulating cell survival and death, which can improve recovery after ischemic neuronal injury. Mitochondrial dynamics are composed of two opposite fission and fusion processes that cooperate to regulate mitochondrial morphology and extend its function, allowing the mitochondrial network to adapt to a cell's needs and external cues [172, 177]. The fusion process involves the elongation of mitochondria by joining and tethering the mitochondria nearby. In contrast, the fission process includes the constriction and cleavage of mitochondria and is mediated by dynamin-related protein 1 (Drp1), a mitochondrial-binding GTPase [178]. Global cerebral ischemia causes a transient increase in Drp1 phosphorylation at serine 616 without notably affecting total protein expression or its phosphorylation at serine 637 in hippocampal CA1 neurons [33]. Drp1 inhibitors reduce the infarct volume in a focal cerebral ischemia model [179]. Mitochondrial fission precedes neuronal death after cerebral ischemia [180]. In addition, few polyphenolic NPs confer neuroprotection by maintaining mitochondrial Ca^2+^ homeostasis. For instance, resveratrol prevents Ca^2+^-induced mitochondrial swelling of neurons after rat brain hypoxic injury [181]. Curcumin prevented okadaic acid-induced memory impairment in mice, where it reduced mitochondrial Ca^2+^ uptake in the hippocampus and cerebral cortex (Fig. **1C**) [182].

Resveratrol stimulates mitochondrial biogenesis by activating the SIRT1-AMPK-PGC1-α axis in cell and animal models of AD, PD, and Down’s syndrome [11], suggesting its neuroprotective role in stroke. Curcumin, a diarylheptanoid found in turmeric (*Curcuma longa*), attenuates neuronal death and prevents cerebral ischemia/reperfusion injury with concomitant increases in mitochondrial mass and expression of the mitochondrial biogenesis regulators NRF-1 and TFAM in rat brains [183]. In addition, flavonoids belonging to different structural classes are potential simulators of mitochondrial biogenesis. The flavanonol dihydromyricetin from Ampelopsis grossedentata protects against neurodegeneration and memory impairment in rats subjected to cerebral hypoxia-ischemia, which increases PGC-1α and TFAM expressions that are responsible for mitochondrial biogenesis in hippocampal neurons [184]. Salidroside, a simple phenolic glucoside from Rhodiola Rosea, protects from hypoxia-induced neurodegeneration and memory impairment and increases PGC-1α, AMPK, and SIRT1 expression and mtDNA content in the rat hippocampus [184]. Regarding plant-derived terpenoids, the monoterpene linalool, commonly found in essential botanical oils, shows protective effects against glutamate toxicity, which increases mitochondrial respiration in HT22 cells [185]. Bicelaphanol A, a dimeric trinorditerpene from Celastrus orbiculatus, increases ATP production in mitochondria and protects from H_2_O_2_-induced mitochondrial stress in PC12 cells [186].

### 3.4 Ferroptosis - Targeting Therapies of NPs in Stroke

Ferroptosis has been implicated in the pathological cell death associated with stroke [187]. During cerebral ischemia, there is rapid ATP loss and uncontrolled ion leakage across the cell membrane due to energy loss, leading to intracellular accumulation of redox‐active iron and ferroptic neuronal death [7, 188]. Ferroptosis is a form of regulated cell death characterized by the iron-dependent accumulation of lipid hydroperoxides to lethal levels. The sensitivity to ferroptosis is tightly linked to numerous biological processes, including amino acid, iron, and polyunsaturated fatty acid metabolism, and the biosynthesis of glutathione, phospholipids, NADPH, and coenzyme Q10 [187]. Increasing evidence demonstrates that NPs, such as saponins, flavonoids, and isothiocyanates, can induce or inhibit ferroptosis. Furthermore, saponins, terpenoids, and alkaloids induce ROS- and ferritinophagy-dependent ferroptosis, whereas flavonoids and polyphenols modulate iron metabolism and NRF2 signaling to inhibit ferroptosis (Fig. **1D**) [189].

## 4 NEUROPROTECTION TARGETING TO APOPTOSIS, AUTOPHAGY, AND NECROPTOSIS IN ISCHEMIC STROKE

### 4.1 Apoptosis - Targeting Therapies of NPs in Stroke

Some specific components of the apoptotic pathway are activated in human ischemic stroke. Apoptotic death plays a significant role in the hypoxic penumbra (oxygen deficiency) and during reperfusion [190]. Apoptosis results from a collapse of cellular infrastructure through internal proteolytic digestion and leads to cytoskeletal disintegration, metabolic derangement, and genomic fragmentation. Apoptosis is mediated through two distinct but interconnected pathways: extrinsic and intrinsic. The activated caspase-8 initiates the extrinsic pathway, then links the intrinsic pathway *via* cleavage of Bid, promotion of Bax recruitment from an inactive cytosolic to the mitochondrial membrane, and regulation of the dynamics of apoptotic pore growth, which causes cytochrome c release and caspase-9 activation, triggering downstream effector caspases-3 and -7 cascade and thereby eventually resulting in apoptosis [191, 192]. Members of the protease caspase family play a significant role in apoptosis and are involved in the initiation, execution, and regulatory phases of the pathway [193].

Convincing proof-of-principle evidence confirms the validity of targeting apoptosis strategies in several animal models [193]. Inhibitors of apoptotic pathways significantly decrease the size of brain infarction. They could be valuable later when given up to 6 h after focal cerebral ischemic insult, which provides significant neuroprotection. Numerous NPs can inhibit or induce apoptosis. The representative examples are apoptolidin, okadaic acid, cerulenin, lactacystin, bryostatin, staurosporine, taxanes, colchicine, laulimalide, geldanamycin, and betulinic acid [194]. The NP apoptolidin targets structurally related macrolides, such as oligomycin and ossamycin. Apoptolidin and some derivatives bind to mitochondrial F0F1-ATPase and induce apoptosis. Major therapeutic targets of apoptosis-based compounds include B-cell lymphoma-2 (BCL2) modulators, caspase activators and inhibitors, p53 modulators, inhibitors of apoptosis proteins, protein-kinase-pathway modulators, and multiple signal-transduction regulators [195], especially caspase-3 is a promising drug target of stroke.

**Fig. (2):**
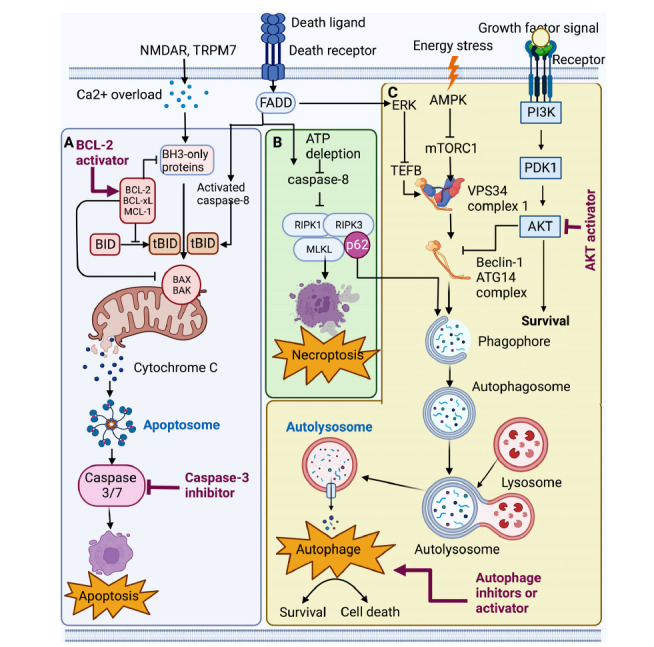
Neuroprotection targeting to signal transduction of apoptosis, necroptosis, and autophagy in ischemic stroke. (**A**) Bcl-2 activators inhibit the intrinsic pathway of apoptosis. In multiple forms of cellular stress, proapoptotic BCL-2 family members Bax and Bak translocate to the mitochondria, then mediate the release of cytochrome *c* in the cytosol and trigger the apoptosome assembly (Apaf-1 and caspase-9) and subsequent activation of caspase-3 and cell death. The extrinsic cell death pathway is that death receptors (CD95, TRAIL-R1/2, and TNF-R1) mediate and recruit adapter proteins FADD and bind to the death effector domain-containing caspase-8 or -10. The activation of caspase-8 mediates cleavage of Bid to tBid or directly activates caspase-3, the executioner enzymes of apoptosis. (**B**) Necroptosis signaling pathway in cerebral ischemic stroke. Under conditions of ATP depletion, inhibition of caspase-8 leads to the formation of a necrosome, including RIPK1, RIPK3, and MLKL, which initiates a downstream signal cascade of necroptosis. (**C**) The process of autophagy in cerebral ischemic stroke. Energy stress rapidly activates the AMPK pathway, inhibits the mTORC1 and VPS34 complex, and finally activates autophagy. In contrast, growth factor signals activate the AKT pathway and mTORC1 through PI3K and then also mTROC1 and inhibit autophagy. At the early stage of cerebral ischemic stroke, oxidative and ER stresses may induce autophagy, preventing necrosis through catabolic energy production and aborting apoptosis *via* eliminating damaged mitochondria. However, at the late stage of cerebral ischemic stroke, a high level of “autophagic stress” leads to massive lysosomal activation and ultimately, cell demise. Depending on the interplays between necrosis, apoptosis, and autophagy, neurons may exhibit mixed features of cell death in ischemic stroke.

Caspases are conservatively evolution cysteine proteases and can be traced back to simpler organisms, such as facultative multicellular organisms like the slime molds (dictyos-telium) that express a paracaspase. Caspase can cleave the target peptide at an aspartic acid position, and most caspase inhibitors contain a strong electrophilic group and bind irreversibly [190]. Caspase inhibitors target apoptotic neurons and expand the time window in animal models of stroke, which decrease the failure of many agents and successfully treat humans in clinical trials for stroke [196, 197]. Calyculin A and okadaic acid are potent inhibitors of protein phosphatases type 1 (PP1) and type 2A (PP2A) and can induce apoptosis through a caspase-3-dependent mechanism in several cancer cell lines [194]. In addition, Kaempferol and quercetin induce caspase-3-dependent apoptosis in various oral cancer cell lines and show cleavage of poly (ADP-ribose) polymerase (PARP) [198]. Carnosic acid modulates Akt/IKK/NF-κB signaling by PP2A and induces intrinsic and extrinsic apoptosis in human prostate carcinoma PC-3 cells (Fig. **2A**) [199].

### 4.2 Necroptosis - Targeting Therapies of NPs in Stroke

Necroptosis is triggered by the activation of death receptors, such as tumor necrosis factor-α (TNF-α). Downstream receptor-interacting protein kinase 1 (RIPK1) is recruited and activated to interact with RIPK3 to initiate the formation of necrosomes and mediate the recruitment and phosphorylation of mixed lineage kinase domain-like protein (MLKL) [200], which form oligomers and then are translocated into the plasma membrane to trigger membrane rupture to mediate necrotic cell death. The mediators of necroptosis, such as RIPK1, RIPK3, and MLKL, have been identified as critical therapeutic targets [200]. For example, the natural product oleanolic acid derivative, 2-cyano-3,12-dioxooleana-1, 9 (11)-dien-28-oic acid (CDDO) has been identified as a novel inhibitor of necroptosis by blocking the death receptor TNFα (Tumor Necrosis Factor) and targeting HSP90 to inhibit the phosphorylation of RIPK1 and RIPK2 in necroptotic cells during cerebral ischemia; the natural product derivative flavanone compound 6E11 is a novel potent small molecular inhibitor of RIPK1-driven necroptosis [201]. Under normal conditions, caspase-8 limits the formation of necrosome [202]. However, during cerebral ischemia, ATP depletion inhibits caspase-8 and triggers necroptosis [7]. Therefore, it is necessary to explore necroptotic target therapy to some extent (Fig. **2B**).

### 4.3 Autophagy - Targeting Therapies of NPs in Stroke

Autophagy is an essential modifier of cell death. Oxidative and endoplasmic reticulum (ER) stresses following cerebral ischemia may induce autophagy, which initially prevents necrosis through catabolic energy production and aborts apoptosis by eliminating damaged mitochondria. However, neuronal autophagy may be further activated under the stress of external ischemia and hypoxia. Neuronal basal autophagy will be further activated by neuronal-induced autophagy *via* the classical PI3K/Akt/mTOR, Beclin-1/LC3A/B-II/Atg5, and AMPK/mTOR/ULK1 pathways [203]. The high level of “autophagic stress” leads to massive lysosomal activation and cell demise. Depending on the interplays between necrosis, apoptosis, and autophagy, neurons may exhibit mixed features of cell death in ischemic stroke [204].

Autophagy may play different roles in ischemic stroke [205] or during subsequent reperfusion [206]. Activation of autophagy [207] and disruption of autophagosome-lysosome fusion [208] may induce ischemic neuronal damage in the hippocampal CA1 region after transient global cerebral ischemia. Autophagy and mitophagy gene BACE1 is found to be dysregulated with similar caspase3 gene expression in post-ischemic rats. The autophagic flux is activated and contributes to ischemic neuronal injury in rats subjected to focal ischemia [209] and cerebral hypoxic ischemia [210, 211]. While autophagy with PER1 or BACE1 provides a protective pathway during periods of injury, it regulates autophagy activity by inducer or inhibitor. In addition, some NPs, through histone post-translational modification, reduce acetyl coenzyme A (AcCoA) levels, inducing the upregulation of autophagy (Fig. **2C**) [212].

Natural polyphenol resveratrol can up-regulate autophagy levels and significantly improve the cognitive levels in model hypoperfusion models; resveratrol can activate Sirt3-mediated autophagy in neuronal HT22 cells and play a protective role in endoplasmic reticulum stress of vascular endothelial cells [213, 214]. By up-regulating autophagy, resveratrol can clear damaged organelles in endothelial cells and inhibit platelet aggregation [214]. Oxymatrine treatment reduced the apoptosis level of I/R rats and increased SIRT1 to up-regulate autophagy levels [203]; ginkgolide K can activate protective autophagy by inducing AMPK/mTOR/ULK1 signaling pathways, thus advancing the proliferation and migration of astrocytes after OGD [215].

## 5 NEUROPROTECTION TARGETING TO ER STRESS AND PROTEASOME IN ISCHEMIC STROKE

### 5.1 ER Stress - Targeting Therapies of NPs in Stroke

**Fig. (3):**
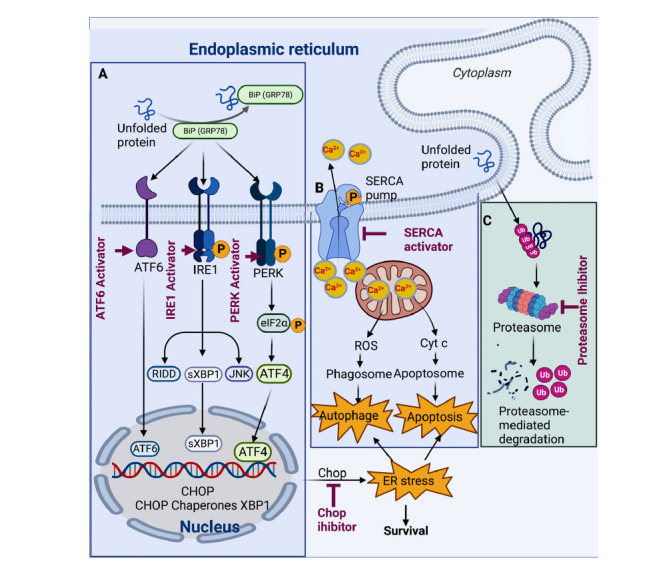
(**A**) Neuroprotection targeted to endoplasmic reticulum stress signaling in ischemic stroke. During ER stress, unfolded proteins accumulate in the ER lumen and sequester BiP from the sensor molecules PERK, IRE1, and ATF6. PERK phosphorylates eIF2α, blocks new secretory protein synthesis, and triggers the integrated stress response through raised ATF4. Activation of IRE1 leads to the generation of active XBP1. Cleavage of ATF6 generates ATF6c. These transcription factors cooperate to induce target genes of the unfolded protein response (UPR). (**B**) In parallel, failure of the sarcoplasmic/endoplasmic reticulum calcium ATPase (SERCA pump) depletes ER calcium and allows calcium influx into the cytosol, which further triggers calcium-induced calcium release *via* ryanodine receptors located in the ER membrane, which leads to calcium depletion from the ER (not shown). In severely affected neurons, raised cytosolic calcium ultimately induces cell death, including apoptosis and autophagy. (**C**) The misfolded proteins are ubiquitinated and eventually degraded by the proteasome.

Several signals can alter ER homeostasis and accumulate misfolded or unfolded proteins in the ER lumen, which causes ER stress response [216]. During cerebral ischemia, energy depletion leads to failure of the sarcoplasmic/endoplasmic reticulum calcium ATPase (SERCA pump) and thus redistribution of ER calcium into the cytosol, releasing ROS and Cytc from mitochondrial and triggering autophagy and apoptosis. In contrast, the inhibition of the SERCA pump induces ER stress or apoptosis [217]. ER stress reduces the protein folding capacity. The unfolded proteins accumulate in the ER lumen. It binds the BiP from the sensor molecules with double-stranded RNA-dependent protein kinase PKR-like ER kinase (PERK), inositol-requiring 1a (IRE1a), activating transcription factor 6 (ATF6). PERK phosphorylates elF2α, blocks the secretory protein synthesis, and triggers the stress response by raising ATF4. Activation of IRE1 leads to the activation of transcription factor X-box binding protein 1 (XBP1); cleavage of ATF6 generates ATF6c. These transcriptional factors ATF4, XBP1, and ATF6c cooperate to induce targeted genes of the unfolded protein response (UPR). The transcription factor CHOP is induced by ATF4 and has widely been regarded as proapoptotic during ER stress [218]. Under severe ER stress, CHOP promotes protein secretion and increases stress intensity, thus inducing cell apoptosis. While in milder ER stress, CHOP does not increase ER stress sufficiently to trigger cell death. Therefore, CHOP expression is cell-type specific, leading to beneficial or harmful effects depending on the extent of ER stress, promoting cell survival or death [219-221]. NP thapsigargin, basiliolide A1, and agelasine B are SERCA inhibitors and profoundly impact Ca^2+^ homeostasis [222, 223]. Resveratrol, withaferin A, and cephalostatin 1 cause the accumulation of misfolded proteins in cancer cells, thus triggering ER stress (Fig. **3**) [223].

### 5.2 Proteasome - Targeting Therapies of NPs in Stroke

Proteasomes in the cytosol and nucleus of mammalian cells are the predominant non-lysosomal machinery for protein turnover. The proteasome is a multicatalytic protease complex that contains the catalytic 20S core, and it is critically involved in the pathophysiological processes in cerebral ischemia. Pharmacological proteasome inhibition in IR injury has recently increased interest [224]. For example, the natural product celastrol is a quinone methide pentacyclic triterpene and inhibits the chymotrypsin-like activity of a purified 20S proteasome [225]; lactacystin is obtained from some streptomyces species and inhibits three chymotrypsin-like, trypsin-like and caspase-like peptidase activities of the proteasome, which induce ER stress and lead to apoptosis [226]. In addition, several flavonoids, such as apigenin, quercetin, myricetin, and kaempferol, have been described as proteasome inhibitors (Fig. **3**).

## 6 NEUROPROTECTION TARGETING TO INFLAMMATION AND BRAIN REPAIR IN ISCHEMIC STROKE

### 6.1 Inflammation - Targeting Therapies of NPs in Stroke

The inflammatory response plays a vital role in the occurrence and development of ischemic stroke, which determines the transformation and prognosis of stroke. After ischemic stroke, damaged neuronal cells become necrotic and release various molecular signals that constitute damage-associated molecular patterns (DAMPs). Endogenous molecules include ATP, high mobility group box 1 (HMGB1), hyaluronic acid, heat shock proteins, and various RNAs [227]. DAMPs act on the related pattern recognition receptors, such as Toll-like receptors (TLRs), to activate downstream signaling pathways, which activate microglia cells and show morphology characteristics within a few minutes after the acute ischemic stroke. Additionally, DAMPs can be recognized by perivascular macrophages, endothelial cells, and neutrophils. The activated microglia and macrophages promote the secretion of various inflammatory factors and elevate leukocyte infiltration, which triggers neuroinflammation. Inflammatory cells can also produce ROS and reactive nitrogen species (RNS), further activating inflammatory cells and entering a vicious cycle [23, 26].

**Fig. (4):**
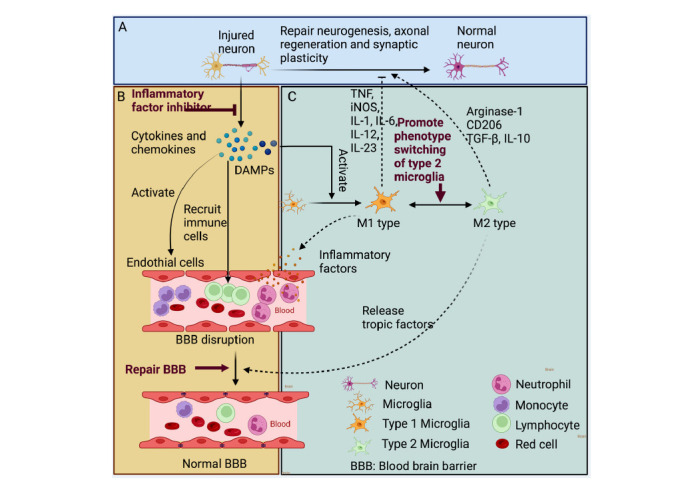
(**A**) In the brain parenchyma, injured cells act as early proinflammatory signals, leading to the production of cytokines and chemokines. While the clearing of dead cells, suppression of inflammation, promotion of neurogenesis, axonal regeneration, and synaptic plasticity are key events in brain repair. (**B**) Ischemic cell death leads to the formation of danger-associated molecular patterns (DAMPs) molecules, which upregulate pro-inflammatory cytokine gene expression, increase leukocyte infiltration, and finally enhance tissue damage and BBB. (**C**) Phenotypic polarization of microglia and macrophages. Microglia and macrophages become polarized towards M1 or M2 phenotypes in ischemic stroke and have distinct roles in restoring the neurovascular network. M1 populations are characterized by the expression of signature proteins, such as TNF, iNOS, and IL-6. They release proinflammatory factors and free radicals that impair brain repair and regeneration. By contrast, M2 populations are characterized by the expression of signature proteins, such as arginase-1, CD206, and IL-10. They improve brain repair and regeneration by increasing phagocytosis, releasing tropic factors, and resolving cerebral inflammation.

The rational administration and combination of NPs can significantly limit the damage of penumbra in ischemic stroke and reduce the neuroinflammatory cascade reactions [23]. Chrysophanol inhibits the production of proinflammatory mediators, cytokines, and ROS by down-regulating the dephosphorylation of drp-1 (s637); it also inhibits the lipopolysaccharide (LPS)-induced inflammation of BV-2 microglia as well as the activation of the NALP3 inflammasome [228]. Celastrol stimulates IL-33 expression and activates the IL-33/ST2 pathway after ischemia by inducing beneficial M2 polarization of microglia/macrophages, thereby suppressing ischemia-induced inflammatory factors expression [229]. Triptolide diminishes neuroinflammation by reducing the increase of proinflammatory cytokines mediated by NF-κB and p38 MAPK pathways in the rat MCAO model [230]. Schisandrin B inhibits the damage of cortical neurons and down-regulates TNF-α, IL-1β, MMP-2, and MMP-9 in ischemic hemispheres. Administration of schisandrin B before the initial injury in the MCAO/R model decreases inflammation and induces microglia activation [231]. Thuja orientalis Semen extract inhibits excessive microglia activation by down-regulating inflammatory responses [232]. Oligomeric proanthocyanidins (OPCs) have inhibitory effects on inflammatory immunity in the brain of rats. Moreover, the hawthorn extract helps relieve I/R-mediated proinflammatory immune responses and increases Foxp3-positive regulatory T Cells (Tregs) in the brain [233]. Ferulic acid treatment markedly reduces MPO-positive cells in the ischemic cortex [234]. After LPS stimulation, NO, PGE2, MCP-1, TNF-a, IL-1β, and IL-6 are highly expressed and are associated with NF-kB activation in BV cells. Whereas Hexane extracts of US (HEUS) inhibit these pro-inflammatory cytokines expression and then protect the brain from acute ischemic damage (Fig. **4**) [235].

### 6.2 Blood Brain Barrier – Targeting Therapies of NPs in Stroke.

The blood-brain barrier (BBB) encompasses the microvasculature of the central nervous system (CNS) and is composed of brain endothelial cells (BECs), pericytes, and astrocyte end-feet embedded in the capillary basement membrane [236]. BECs are non-fenestrated and possess extensive tight junctions (TJs) and a higher metabolic activity owing to fourfold higher cytoplasmic mitochondrial volume than other non-BBB endothelial cells [237, 238]. BECs are susceptible to apoptosis and tight junction disruption in response to hypoxic or ischemic conditions [239]. During cerebral ischemic stroke, tight junction proteins are degraded in BECs, then BBB disruption and permeability are increased [238]. Increased BBB permeability precedes and facilitates the infiltration of immune cells into the parenchyma, triggering inflammation by intracellular and extracellular signaling mechanisms that lead to neuronal death or dysfunction. The dysfunction or alteration of endothelial cells may precede neuronal death, which thus makes the BBB and/or the endothelial layer an exciting target for therapeutic intervention in stroke [238, 240]. In addition, BECs secrete neurotrophins, such as brain-derived neurotrophic factor (BDNF), insulin-like growth factor 1 (IGF1), and vascular endothelial growth factor (VEGF), to maintain normal brain homeostasis, suggesting that neurotrophic factors are a potent drug candidate for stroke therapy [238, 241]. However, although BBB is not fully intact in humans after ischemic stroke, most drug categories do not readily cross BBB. The breakdown of BBB is heterogeneous among individuals, and an optimal therapy would readily penetrate the BBB and the brain to reach distal aspects of an evolving infarction zone [196, 242].

After ischemic stroke, the expression of intercellular adhesion molecule-1 (ICAM-1), vascular cell adhesion molecule 1 (VCAM1), integrin, and E-selectin is increased, which promotes peripheral immune cell penetration into the brain tissue and aggravates nervous system inflammation. Within 2 h of cerebral ischemia, MMPs degrade TJPs in the vascular lumen, disrupt the junctions between endothelial cells and pericytes, and increase BBB permeability. The level of MMP-9 is related to the severity and prognosis of initial cerebral ischemia [243]. Natural medicine protects the BBB, which looks like a promising strategy for treating ischemic stroke. Salvianolic acid crosses the BB and neutralizes free radicals; Sal A decreases the expression of ICAM-1 and inhibits granulocyte adhesion to brain microvascular endothelial cells in ischemia and hypoxia. Tan IIA restores the expression of major TJPs in a dose-dependent manner and effectively attenuates the extent of brain oedema formation by down-regulating ICAM-1 and MMP9 [244, 245]. Schisandrin B inhibits the damage of cortical neurons, which is associated with the downregulation of TNF-α, IL-1β, MMP-2, and MMP-9 in ischemic hemispheres [231]. Moreover, natural medicine, such as Tanshinone IIAA [245], Z-ligustilide [114], Cerebralcare Granule^®^ [246], Tongxinluo [247], Uncaria Sinensis [244], *etc*., protect the brain from BBB disruption *via* alleviating inflammatory response or brain edema after stroke (Fig. **4**).

## CONCLUSION AND PERSPECTIVES

Stoke is one of the most common causes of disability and death worldwide, which seriously endangers human health, bringing a heavy burden to society and families. Until now, there has been no effective therapy for treating cerebral stroke. Intravenous and endovascular thrombectomy is utilized clinically. However, their use is restricted due to short therapy time windows and the risk of bleeding. NPs have been used to treat diseases, such as nervous disorders, cancer, and infectious diseases. Nowadays, many attempts have been made to explore the neuroprotective roles of NPs in ischemic stroke. Therefore, we firstly reviewed the pharmacological properties of various NPs. Then, we elaborated on the pathophysiology of cerebral ischemic stroke, including many processes, such as intracellular calcium imbalance, toxic effects of excitatory neurotransmitters, inflammatory reactions, and destruction of the blood-brain barrier (BBB) [248, 249].

However, several questions remain unclear and need to be addressed in future studies. Firstly, how to interpret and confirm the precise mechanisms of NPs in ischemic stroke. Different types of NPs have different structures, while the structure-activity relationship of the NPs is mainly related to the basic nucleus of the compound, the number and position of double bonds, and the position of functional groups. Using network pharmacology methods, the structure and function of NPs can be further analyzed. Then, animal models and cell experiments can be used to verify the neuroprotective effects of NPs on cerebral ischemic stroke through *in vivo* and *in vitro* studies. Secondly, how to comprehensively evaluate the therapeutic effect of NPs on cerebral stroke. In addition to the effects on the acute phase of stroke, some NPs also have significant therapeutic effects on cognitive behavior in the chronic phase. Thirdly, how to physically or chemically modify the NPs, increase their entry into the BBB, improve their bioavailability, and reduce their toxic and side effects. In recent years, nanocarrier-based biomedicine has been developed and aimed at improving treatment paradigms. Nanogels serve as multipurpose and constructed vectors formed *via* intramolecular cross-linking to generate drug delivery systems, which is attributed predominantly to their satisfactory biocompatibility, bio-responsiveness, high stability, and low toxicity [132]. Fourthly, how to use NPs to treat comorbidity in stroke patients. As is known, many stroke patients are aging with comorbidities, such as hypertension, diabetes, *etc*., which affect the treatment effect of stroke. Accordingly, there are also some primitive inflammatory pathologies, such as vasculitis as a cause of the stroke [250], as well as some systemic inflammatory responses in patients with ischemic stroke undergoing endovascular treatment [251]. Furthermore, regarding acute neurovascular accidents and neurological sequelae in patients with COVID-19, SARS-CoV-2 can induce a systemic inflammatory response and a hypercoagulable state and lead to direct vascular endothelial damage [252]. Older individuals with COVID-19 are at higher risk for neurovascular events. In a retrospective cohort of 221 subjects, eleven (5%) underwent ischemic stroke, one (0.5%) cerebral hemorrhage, and one (0.5%) cerebral venous thrombosis [252, 253]. Chinese herbal medicine (CHM) as adjunctive therapy could improve overall survival. At the same time, in future studies, using aging, vasculitis, or COVID-19 models to examine the effects of NPs in stroke is highly recommended for realizing their clinical application. Fifthly, how to prospectively develop novel, efficacious, and safe neuroprotective NPs with sex-specificity in stroke incidents. There is ample evidence that the pathophysiology of stroke is based on sex differences [254]. Moreover, epidemiological studies have also shown that stroke is more common among men, but women are more severely ill worldwide [255]. Therefore, it should be necessary to develop sex-specific pharmacologic strategies for stroke prevention and treatment. Finally, how to evaluate the different preventive or therapeutic roles of NPs in ischemic stroke. Most NPs have therapeutic potential based on recent experimental findings. In most studies, NPs were used after the MCAO model, and their therapeutic roles were analyzed. While some NPs are preventive, and their roles are observed using a preconditioning stroke model. For example, resveratrol preconditioning 14 days before MACO extended the window of ischemic tolerance and induced neuroprotection in the mouse brain [62, 65]. In addition, some NPs are both preventive and therapeutic. For example, ligustilide ameliorated not only neuronal injury in ischemic stroke [110-113] but also intranasal delivery of ligustilide enhanced protection against ischemic injury, indicating its prophylactic potential in the population at high risk of stroke [114]. Therefore, the preventive or therapeutic effects of NPs need to be evaluated according to dosing before or after the MCAO model. In summary, NPs would be very valuable when seeking novel therapeutic agents for stroke.

## Figures and Tables

**Table 1:** NPs-derived flavonoids, phenols, terpenes, lactones, quinones, alkaloids, glycosides, and phenylpropanoids as the neuroprotective targets in ischemic stroke.

**S. No.**	**Category**	**Compounds and Structure**	**Source**	**Signal Pathways**	**Targets**	**References**
1	Flavonoids	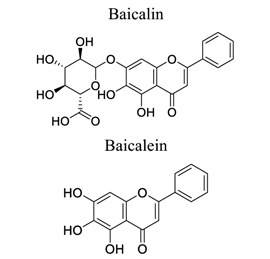	*Scutellaria * *baicalensis*	Inhibiting TLR2/4, NF-κB p65, COX-2 expression, phosphorylation of CaMKII. Involved in PI3K/Akt and PTEN pathways	Anti-oxidation. Anti-apoptosis. Anti-inflammation. Anti-excitotoxicity. Protecting mitochondrial function. Promoting neuronal factors and neurogenesis	[34-42]
2		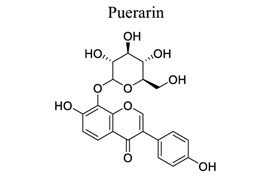	*Puerariae * *lobata*	Activating ERK/HIF-1a-, PI3K/AKT/mTOR/HIF-1a-dependent pathways	Anti-apoptosis. Anti-inflammation.	[43-45]
3		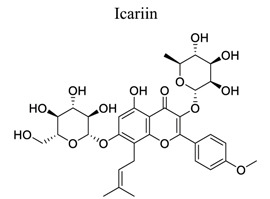	*Berberidaceae,* *Epimedium* L.	Increasing VEGF and BDNF expression through PI3K and ERK1/2 pathways	Promoting angiogenesis and neurogenesis. Anti-inflammation. Antioxidant.	[44]
4		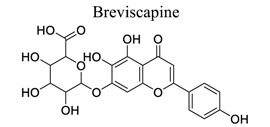	*Erigerin * *breviscapus*	Counteracting autophagy inducer Tat-Beclin-1. Inhibiting inflammatory cytokines and NF-κB.	Attenuating autophagy. Anti-oxidation. Anti-inflammation	[46, 47]
5		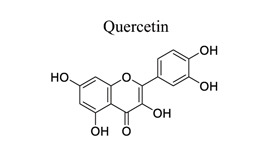	*Cranberries, grapes, apples, cherries, onions, peppers, black* and *green tea, and wine*	Activating mitochondrial BK-Ca channels in endothelial cells.	Reducing calcium overload, BBB permeability, and brain water content. Anti-oxidation. Anti-apoptosis. Anti-inflammation	[48, 49]
6		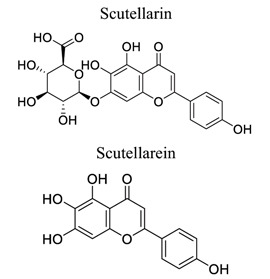	*Erigeron * *breviscapus* Hand Mazz	Attenuating NF-κB, Notch-1, NICD, RBP-JK and Hes-1 in activated microglia. Regulating expression of NOS isoforms and angiogenic molecules.	Activating microglia. Reducing BBB permeability	[50-52]
7	Flavonoids	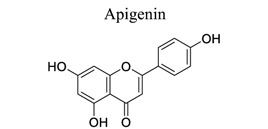	*Dietary, plant, vegetables and fruits*	Affecting caveolin-1/VEGF, Bcl-2, caspase-3, beclin-1, and mTOR; Activating PI3K/Akt/Nrf2.	Inhibiting apoptosis, autophagy, and inflammation. Promoting recovery of tissue structure.	[53, 54]
8		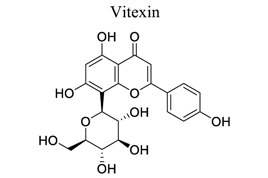	*Hawthorn, * *passionflower, bamboo leaves, chasteberry*	Regulating mTOR/Ulk1, mitogen-activated protein kinase, apoptosis signals. Attenuating HIF-1α and VEGF expression.	Reversing autophagy dysfunction; Increasing Bcl-2/Bax ratio. Improving BBB integrity and neurobehavioral outcomes.	[55, 56]
9	Phenols	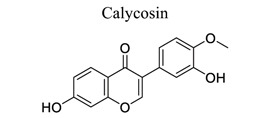	*Radix astragali Mongolici*	Suppressing toll-like receptors, PI3K-AKT, TNF, MAPK, VEGF, and calpain activation. Increasing TRPC6 and P-CREB expression.	Improving endothelial cell proliferation and growth, inflammatory development, and cellular metabolism. Modulating intracellular Ca^2+^ levels.	[56, 61]
10		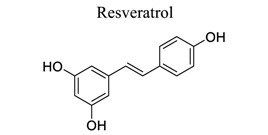	*Grapes* and *red wine*	Activating Nrf2, TLR4/NF-κB/ STAT cascades, as well as SIRT1-regulated pathways	Anti-oxidation. Anti-inflammation. Mimicking ischemic preconditioning.	[62-65]
11		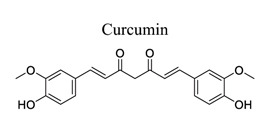	*Curcuma longa*	Protecting BBB. Inhibiting xanthine dehydrogenase/xanthine oxidase (XD/XO) conversion and O2 production. Inhibiting NF-κB and NLRP3 inflammasome.	Anti-oxidation. Anti-inflammation. Inhibiting mitochondrial apoptosis. Reducing M1 microglial activation and pyroptosis.	[42, 66-69]
12		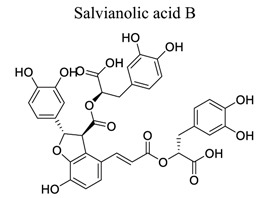	*Salvia * *miltiorrhiza* Bge.	Inhibiting the decrease in SOD, GSH, and ATP levels and the increase in MDA and LA levels.	Anti-oxidation. Inhibiting regional cerebral blood flow (rCBF) and platelet aggregation.	[70-72]
13		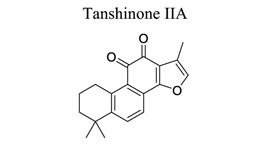	*Salvia * *miltiorrhiza*	Modulating MAPK pathways. Inhibiting MMP-9. Increasing Bcl-2. Decreasing Bax and cleaved caspase-3 levels	Anti-inflammation; Reducing BBB damage. Inhibiting glutamate-induced apoptosis.	[73-75]
14		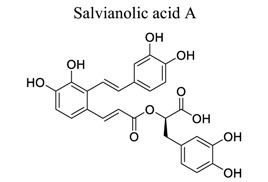	*Salvia * *miltiorrhiza* Bunge	Inhibiting AKT/FOXO3a/BIM. Involved in Drd2/Cryab/NF-κB. Compromising GSK3β/Cdk5 activity. Enhancing β-catenin/DCX and Bcl-2 levels. Suppressing Src signaling pathway.	Anti-apoptosis. Relieving cognitive disorder. Preserving BBB integrity. Promoting neurogenesis. Preventing cerebrovascular endothelial injury.	[76-79]
15	Phenols	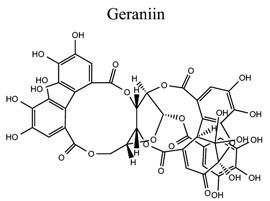	*Phyllanthus amarus*	Regulating the Nrf2/HO-1 pathway.	Suppressing oxidative stress and neuronal apoptosis.	[80]
16		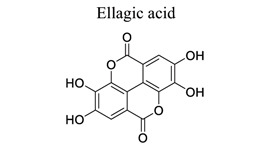	*Raspberry, red fruits*	Regulating Bcl-2/Bax ratio.	Anti-inflammation. Anti-apoptosis	[81]
17	Terpenes	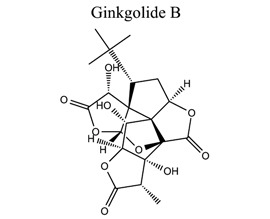	*Ginkgo biloba leaves*	Inhibiting creatine kinase B and regulating CCT/TRiC-SK1 axis. Activating EP4, PKA, Akt, ERK1/2, as well as Src-mediated transactivation of EGFR. Increasing BDNF and EGF.	Pro-angiogenic activity. An antagonist of platelet-activating factor (PAF). Inhibiting apoptosis. Modulating microglia polarization. Promoting proliferation and differentiation of neural stem cells.	[85-89]
18		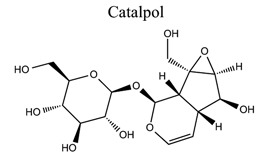	*Radix* * rehmanniae*	Activating GLP-1 receptor/β-endorphin and HIF-1α/VEGF; Increasing brain levels of EPO and VEGF without worsening BBB edema.	Improving brain angiogenesis and ameliorating the edema. Antioxidation; Anti-Inflammation; Anti-apoptosis.	[90-93]
19		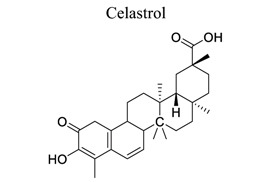	*Tripterygium wilfordii Hook*	Targeting HSP70, NF-κB p65, and binding to HMGB1; Promoting an IL-33/ST2 axis. Targeting the HIF-1*α*/PDK1 axis	Anti-inflammation. Anti-oxidation. Mediating microglia/ macrophage M2 polarization. Inhibiting glycolysis.	[94-97]
20		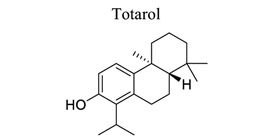	*Podocarpus totara*	Activating Akt/HO-1 pathway.	Reducing oxidative stress.	[98]
21		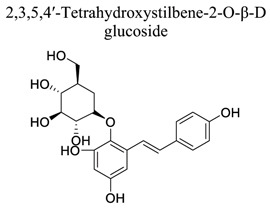	*Polygonum * *multiflorum*	Involved in the JNK, SIRT1, and NF-κB. Inhibiting ROS/RNS. Upregulating VEGF, angiopoietin 1, CD31, and Glutamate Transporter 1 expression. Targeting 5-HT/5-HTR pathways.	Anti-oxidation. Anti-apoptosis. Promoting angiogenesis Anti-excitotoxicity	[99-102]
22	Lactones	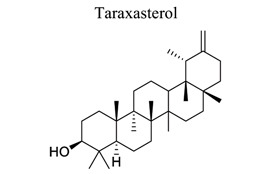	*Taraxacum * *officinale*	Regulating the Nrf2 signal pathway.	Anti-oxidation. Anti-apoptosis.	[106]
23		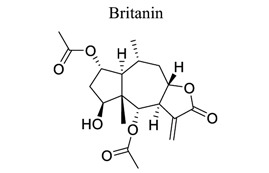	*Inulae flos*	Inducing the Nrf2 pathway.	Anti-oxidation.	[104]
24		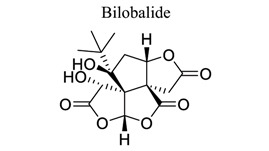	*-*	Inhibiting pro-inflammatory mediator production. Down-regulating JNK1/2 and p38 MAPK activation.	Anti-inflammation. Anti-excitotoxicity. Anti-oxidation. Regulating mitochondrial biosynthesis	[107-109]
25		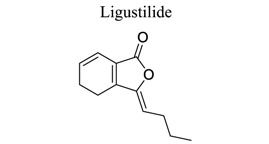	*Rhizoma Chuanxiong*	Inhibiting TLR4/peroxiredoxin 6. Activating the PI3K/Akt, PINK1/Parkin, AMPK pathway, Nrf2 and HSP70, Drp1 signaling.	Anti-inflammation. Anti-apoptosis. Activating autophagy. Anti-mitochondrial dysfunction. Mitigating BBB disruption.	[110-115]
26	Quinones	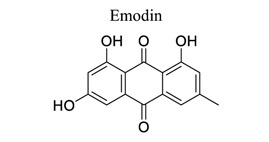	*Dried rhizomes* and *the root of the Rhizoma Polygoni * *Cuspidati*	Activating ERK-1/2, PI3K/AKT/mTOR, and NF-κB pathway, as well as the activin A pathway. Suppressing CAMKII/DRP1-mediated mitochondrial fission.	Anti-apoptosis. Anti-oxidation. Anti-excitotoxicity. Anti-inflammation. Anti-mitochondrial dysfunction.	[119-121]
27	Alkaloids	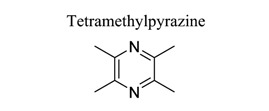	*Ligusticum walliichi (Chuanxiong)*	Activating BDNF/Akt/CREB and JAK/STAT. Suppressing HIF-1α, TNF-α, and caspase-3. Upregulating MCPIP1. Activating PI3K. Elevating Nrf2/HO-1.	Promoting neurodegeneration. Anti-apoptosis. Preserving BBB integrity. Promoting migration of neural precursor cells. Anti-inflammation.	[125-128]
28		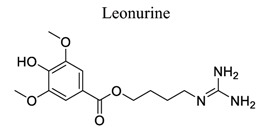	*Herba leonuri*	Inhibiting mitochondrial ROS production and ATP biosynthesis. Regulating HDAC4/NOX4/MMP-9 tight junction pathway. Activating Nrf2 pathway.	Anti-oxidation. Regulating mitochondrial dysfunction. Protecting BBB integrity.	[129-131]
29		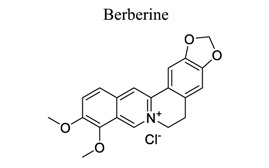	*Coptis chinensis (Huang lian)*	Activating PI3K/Akt, JAK2/STAT3, SIRT1/FoxO3α, AMPK signaling pathways. Suppressing p53/cyclin D1 and HMGB1/TLR4/NF-κB, GRP78 and CHOP. Enhancing beclin-1 and p62 expression. Downregulating CNPY2	Anti-oxidation. Anti-apoptosis. Anti-inflammation. Suppressing ER stress; Promoting autophagy. Regulating periphery immune system. Modulating microglial polarization	[132-135]
30	Glycosides	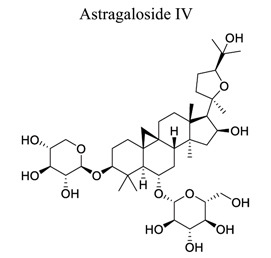	*Radix astragali*	Inhibiting LC3 II/LC3 I ratio and activating p62. Inhibiting calcium-sensing receptors. Mediating JAK2/STAT3 signals.	Anti-apoptosis. Promoting autophagy. Anti-oxidation. Anti-inflammation. Reducing BBB permeability.	[70, 138-141]
31		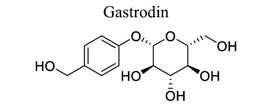	*Gastrodia elata*	Inhibiting NMDA receptor.	Antagonizing glutamate excitotoxicity. Anti-inflammation.	[142]
32		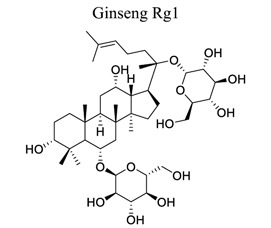	*Panax * *notoginseng* and *ginseng*	Activating Akt, Nrf2/HO-1, PPARγ/HO-1. Activating MAPK, caspase 3/ROCK1/MLC, HMGB1/TLR2/4/9 and NF-κB signal pathways.	Anti-inflammation. Anti-oxidation. Anti-apoptosis. Anti-ER stress	[143]
33		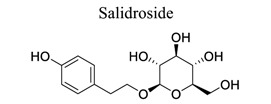	*Rhodiola rosea*	Suppressing TNF-α release, Bax/Bcl-2 ratio, and inflammatory cytokines.	Scavenging free radical. Inhibiting inflammation and apoptosis	[144]
34	Phenylpropanoids	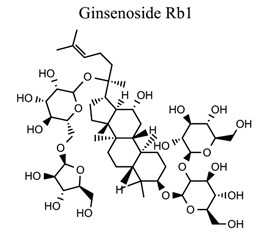	*Panax * *ginseng*	Inhibiting mitochondrial complex I.	Avoiding astrocyte activation.	[146]
35		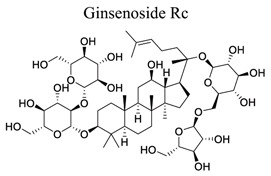	*Panax * *ginseng*	SIRT1 restoration-mediated reduction of PGC1α acetylation.	Inducing mitochondrial biosynthesis.	[147]
36		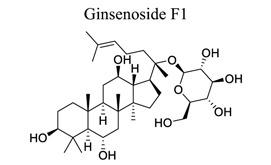	*Panax * *ginseng*	Activating IGF-1/IGF1R pathway.	Promoting angiogenesis.	[148]
37	Phenylpropanoids	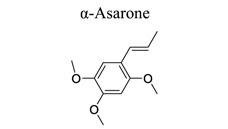	*Acorus * *gramineus*	Activating PPARγ-GLT-1 signaling. Decreasing GFAP, Iba-1, and LC3II/LC3I. Increasing p62 expression.	Enhancing glutamate transport. Alleviating dysmyelination and autophagy.	[149, 150]
38		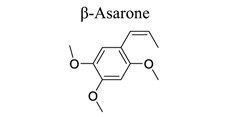	*Acorus * *tatarinowii * *Schott*	Attenuating Beclin-1; Decreasing [Ca^2+^] (i) and increasing MMP. Modulating JNK, p-JNK, Bcl-2, and Beclin 1.	Anti-autophagy Anti-oxidation.	[151-153]
39		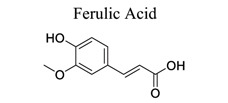	*Feruloyl * *esterase*	Inhibiting p38 MAPK. Enhancing GABA(B1) receptor expression	Anti-apoptosis.	[154]
40		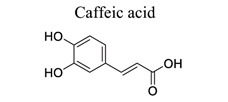	*Propolis*	Inhibiting NF-κB, NOS, and caspase 1, inhibiting Ca^2+^-induced cytochrome c release.	Anti-inflammation Anti-apoptosis Anti-oxidation.	[155]

## LIST OF ABBREVIATIONS

AIS: Acute Ischemic Stroke
BBB: Blood-brain Barrier
ER: Endoplasmic Reticulum
ICH: Intercellular Hemorrhagic
IR: Ischemic Reperfusion
MCP: Momordica Charantia Polysaccharide
MMPs: Metalloproteinases
NO: Nitric Oxide
NPs: Natural Products
ROS: Reactive Oxygen Species
tPA: Tissue Plasminogen Activator
